# Differential diagnosis and cause-specific treatment during out-of-hospital cardiac arrest: a retrospective descriptive study

**DOI:** 10.1186/s13049-023-01080-2

**Published:** 2023-04-11

**Authors:** Elina Heikkilä, Milla Jousi, Jouni Nurmi

**Affiliations:** Department of Emergency Medicine and Services, University of Helsinki and University Hospital, Helsinki, Finland

**Keywords:** Air ambulances, Helicopter emergency medical service, Emergency medical service, Critical care, Out-of-hospital cardiac arrest

## Abstract

**Background:**

The cardiopulmonary resuscitation (CPR) guidelines recommend identifying and correcting the underlying reversible causes of out-of-hospital cardiac arrest (OHCA). However, it is uncertain how often these causes can be identified and treated. Our aim was to estimate the frequency of point of care ultrasound examinations, blood sample analyses and cause-specific treatments during OHCA.

**Methods:**

We performed a retrospective study in a physician-staffed helicopter emergency medical service (HEMS) unit. Data on 549 non-traumatic OHCA patients who were undergoing CPR at the arrival of the HEMS unit from 2016 to 2019 were collected from the HEMS database and patient records. We also recorded the frequency of ultrasound examinations, blood sample analyses and specific therapies provided during OHCA, such as procedures or medications other than chest compressions, airway management, ventilation, defibrillation, adrenaline or amiodarone.

**Results:**

Of the 549 patients, ultrasound was used in 331 (60%) and blood sample analyses in 136 (24%) patients during CPR. A total of 85 (15%) patients received cause-specific treatment, the most common ones being transportation to extracorporeal CPR and percutaneous coronary intervention (PCI) (n = 30), thrombolysis (n = 23), sodium bicarbonate (n = 17), calcium gluconate administration (n = 11) and fluid resuscitation (n = 10).

**Conclusion:**

In our study, HEMS physicians deployed ultrasound or blood sample analyses in 84% of the encountered OHCA cases. Cause-specific treatment was administered in 15% of the cases. Our study demonstrates the frequent use of differential diagnostic tools and relatively infrequent use of cause-specific treatment during OHCA. Effect on protocol for differential diagnostics should be evaluated for more efficient cause specific treatment during OHCA.

## Background

During cardiopulmonary resuscitation (CPR), the resuscitation guidelines recommend considering the potential causes or aggravating factors following the 4 Hs and 4 Ts memory aid (hypovolemia, hypo- or hyperthermia, hypo- or hyperkalaemia, hypoxia, tension pneumothorax, tamponade, toxins and thrombosis). Return of spontaneous circulation (ROSC) during out-of-hospital cardiac arrest (OHCA) might be more likely if considerable underlying pathology is identified and treated [1–4].

In theory, differential diagnostics during CPR can be facilitated with a point-of-care ultrasound (POCUS) examination and laboratory analyses. These diagnostic strategies could lead to patient-specific treatment plans and, consequently, to better chances of ROSC [1–4]. The feasibility of diagnostic tools during CPR has been demonstrated [5–7]. However, identification of certain underlying pathologies with POCUS might not be possible due to changes caused by the arrest. [8, 9] Further, the interpretation of point-of-care (POC) laboratory results may pose significant challenges, and the appropriate treatment can vary [10, 11]. A recent study on POC laboratory analyses during OHCA found a small improvement in survival among patients who underwent POC laboratory analyses during resuscitation. [10]

In order to develop better protocols for differential diagnostics and cause-specific treatment, more information is needed on the frequencies of different reversible causes of OHCA and the use of diagnostic tools. The aim of this study was to examine how often a POCUS examination and blood sample analysis were performed and to identify any cause-specific treatment administered during OHCA.

## Methods

### Study desing

We performed a retrospective descriptive study based on the Helicopter Emergency Medical Service (HEMS) database and patient records. No portion of the study had influence on patient treatment and therefore patient consent was neither required nor acquired. The reporting of the study followed the STROBE guidelines [12]. The study was approved by the ethics committee of Helsinki University Hospital (HUS/3115/2019) and permission was granted by the hospital district (HUS/280/2019).

### Setting

The study was conducted in Southern Finland in an area covered by multiple emergency medical service (EMS) system providers and one physician-staffed HEMS unit. The HEMS unit operates in a geographical area of 20,000 km.^2^ inhabited by 1.3 million people. Annually, the HEMS unit receives, on average, 3,000 primary missions and encounters1,200 patients. All missions are registered in the national HEMS database [13].

Generally, an advanced life support (ALS) EMS unit, an EMS medical supervisor, a fire rescue unit and a HEMS unit are dispatched simultaneously for every witnessed OHCA case. For unwitnessed OHCA cases HEMS unit is dispatched if the patient is known to be alive within 20 min prior to the call. Patients are treated according to the European Resuscitation Council (ERC) ALS algorithm. Since the team includes many ALS-capable professionals, the HEMS unit can primarily focus on performing differential diagnostics and considering possible underlying causes and treatment options while the team runs the routine ALS protocol. During CPR, the HEMS physician performs differential diagnostics using POCUS and/or by performing a blood sample analysis when necessary and they subsequently decide whether to provide cause-specific treatment or withdraw CPR. Rapid transportation to hospital for extracorporeal CPR (ECPR) and coronary angiography is provided for patients with recurrent or refractory ventricular fibrillation (VF) if they are less than 70 years old and without major medical diseases in cases of witnessed cardiac arrest (CA) and an EMS delay of less than 10 min. These criteria are regional and derived from international CPR guidelines [1–4]. The physician generally escorts the patient to hospital since the patient might be in the need of advanced treatment by the HEMS crew.

### Participants

The OHCA cases between 1 January 2016 and 10 July 2019 identified in the HEMS database were included in the study if CPR was ongoing when the HEMS crew encountered the patient. The exclusion criteria were ROSC before the HEMS crew’s arrival, valid ‘do not attempt to resuscitate’ (DNAR) order and traumatic CA. Trauma patients were excluded, since the treatment follows a specific algorithm. We gathered data on patient characteristics, OHCA events (delays, initial rhythm, witness status, bystander CPR and presumed aetiology), CPR details (airway management, medication, use of external chest-compression device and defibrillation), use of POCUS or blood sample analysis during CPR, including the findings documented in the patient records, and the cause-specific treatment provided during CPR. Definitions by the Utstein guidelines for reporting OHCA were followed [14].

### Study procedures

Because a cause-specific treatment is difficult to define due to limited literature, we considered all interventions other than those included in normal ALS (airway management, ventilation, chest compression, defibrillation, adrenaline [epinephrine], amiodarone and intravenous fluids without particular fluid resuscitation) as cause-specific treatments in this study. These were correction of electrolyte abnormalities or acid-base balance disorders, fluid treatment in case of suspected hypovolaemia, glucagon administration, glucose infusion, thoracostomy, pericardiocentesis, thrombolytic therapy and blood product transfusion. Crystalloids were considered a cause-specific treatment if fluid resuscitation or fluid bolus was given as a treatment for possible reversible cause of cardiac arrest and mentioned in the medical records as a treatment for hypovolemia. Transporting patients with ongoing resuscitation managed by Lund University Cardiopulmonary Assist System (LUCAS) to extracorproreal cardiopulmonary resuscitation (ECPR) was also considered a cause-specific treatment in cases of suspected coronary thrombosis or drowning. Coronary angiography is always provided for suspected coronary thrombosis. Only the treatment provided during CPR or transient ROSC (less than 20 min) were taken into account in the study. Treatment of asphyxia and airway obstruction were not considered cause-specific treatments in this study, since airway management and ventilation with oxygen are already included in the general ALS algorithm. The appropriateness of the interventions was not evaluated. Both POC blood sample analyses device (i-STAT analyser, Abbott, IL, USA) and ultrasound device (V-scan and V-scan dual probe, GE Healthcare, Chicago, USA, were always carried by the HEMS unit and used by the consideration of a physician.

### Statistical analyses

The data were analysed using IBM SPSS 25 Windows (IBM SPSS Statistics 25, IBM Corporation, Armonk, NY, USA). Continuous variables are reported as median (interquartile range) and categorical variables as count (%). The 95% confidence interval was calculated for the proportions of diagnostic procedures and the cause-specific therapy. The specific treatment given in different groups according to diagnostic tools used were compared using Fisher’s exact test.

## Results

**Fig. 1:**
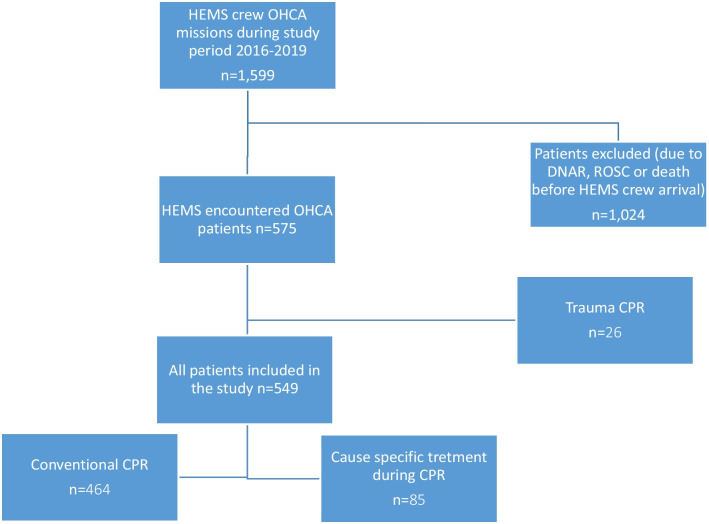
Selection of patients. HEMS, helicopter emergency medical service; OHCA, out-of-hospital cardiac arrest; DNAR, do not attempt to resuscitate; ROSC, return of spontaneous circulation; CPR, cardiopulmonary resuscitation

During the study period, the HEMS unit was dispatched to 1,599 OHCA cases. In 549 of the cases (all of which were included in the study; see Fig. 1), the patients were undergoing CPR when the HEMS unit arrived. Of the 549 patients 414 (75%) were male, 350 (65%) were witnessed arrest and the initial rhythm was VF in 168 (30%) of the cases. The characteristics of the patients are shown in Table 1.

**Table 1:** Patient characteristics of the study population

	All patients n = 549	No cause-specific treatment n = 464	Cause-specific treatment n = 85
Number of males	414 (75%)	346 (74%)	68 (80%)
Age, years	66 (56–74)	67 (58–75)	62 (52–70)
*Witness status*			
Witnessed	350 (63%)	311 (67%)	39 (46%)
Non-witnessed	114 (20%)	101 (21%)	13 (15%)
Witnessed by the EMS unit	85 (15%)	52 (11%)	33 (39%)
*Response*			
Bystander CPR	296 (53%)	264 (56%)	32 (37%)
EMS delay (minutes)	7 (4–10)	7 (5–10)	6 (0–9)
HEMS delay (minutes)	20 (15–25)	20 (15–25)	19 (13–26)
*Cause of cardiac arrest*			
Medical	509 (92%)	427 (92%)	82 (96%)
Overdose	13 (2%)	13 (3%)	0 0
Drowning	8 (1%)	6 (1%)	2 (2%)
Asphyxia	19 (3%)	18 (3%)	1 (1%)
*Initial rhythm*			
PEA	238 (43%)	201 (43%)	37 (43%)
Asystole	138 (25%)	127 (27%)	11 (12%)
VF	168 (30%)	133 (28%)	35 (41%)
VT	4 (1%)	2 (1%)	2 (2%)

Categorical variables are presented as frequencies (percentages), and continuous variables are presented as medians (interquartile ranges). The delays were measured from the dispatch call.

*EMS* Emergency medical service, *HEMS* Helicopter emergency medical service, *CPR* Cardiopulmonary resuscitation, *PEA* Pulseless electrical activation, *VF* Ventricular fibrillation, *VT* Ventricular tachycardia.

Both POCUS and blood sample analyses were performed on 98 (18%, 95% CI: 15–21) patients (Table 2). Of all the patients, 85 (15%, 95% CI: 13–19) received cause-specific treatment (Table 2). In the ECPR treatment group, 30 were due to suspected coronary thrombosis and 2 were due to drowning (Table 3).

**Table 2:** Frequencies of different diagnostic tools and point-of-care ultrasound (POCUS) findings

	All patients N = 549	With no cause specific treatment N = 464	With cause spesific treatment N = 85
*Differential diagnosis*			
No differential diagnostic tools	180 (33%)	155 (33%)	25 (29%)
POCUS alone	233 (42%)	208 (45%)	25 (29%)
Blood sample analysis alone	38 (7%)	26 (6%)	12 (14%)
POCUS + blood sample analysis	98 (18%)	75 (16%)	23 (27%)
*POCUS findings*			
Myocardial contraction	68 (12%)	50 (11%)	18 (21%)
Pericardial effusion	17 (3%)	12 (2%)	5 (6%)
Right ventricle dilation	13 (2%)	8 (1%)	5 (6%)
Hypovolemia	5 (1%)	3 (1%)	2 (2%)
Other findings	35 (6%)	30 (6%)	5 (6%)

*POCUS* Point-of-care ultrasound

Categorical variables are presented as frequencies (percentages)

**Table 3:** Administration of cause-specific treatments during cardiopulmonary resuscitation (n = 85)

Cause-specific treatment	Cases (n = 85)
ECPR	32 (37%)
Thrombolysis	23 (27%)
NaHCO_3_	17 (20%)
Calcium	11 (12%)
Crystalloids	10 (11%)
Red blood cells	5 (5%)
Potassium	4 (4%)
Pericardiocentesis	3 (3%)
Plasma	2 (2%)
Glucose	2 (2%)
Magnesium	1 (1%)
Thoracostomy	1 (1%)

*ECPR* Extracorporeal cardiopulmonary resuscitation, *NaHCO*_*3*_ Sodium bicarbonate

Of the patients without diagnostic tool used, only POCUS used, only blood sample analysis and both used 25/180 (29%), 25/223 (11%), 12/38 (32%) and 23/98 (23%) received specific treatment, respectively. The frequency of cause-specific treatment was significantly higher within the patients who underwent both a POCUS and blood sample analysis (n = 23/98, 23%) than in the patients examined using only one or neither of the methods (n = 62/451, 14%), p = 0.02.

**Fig. 2:**
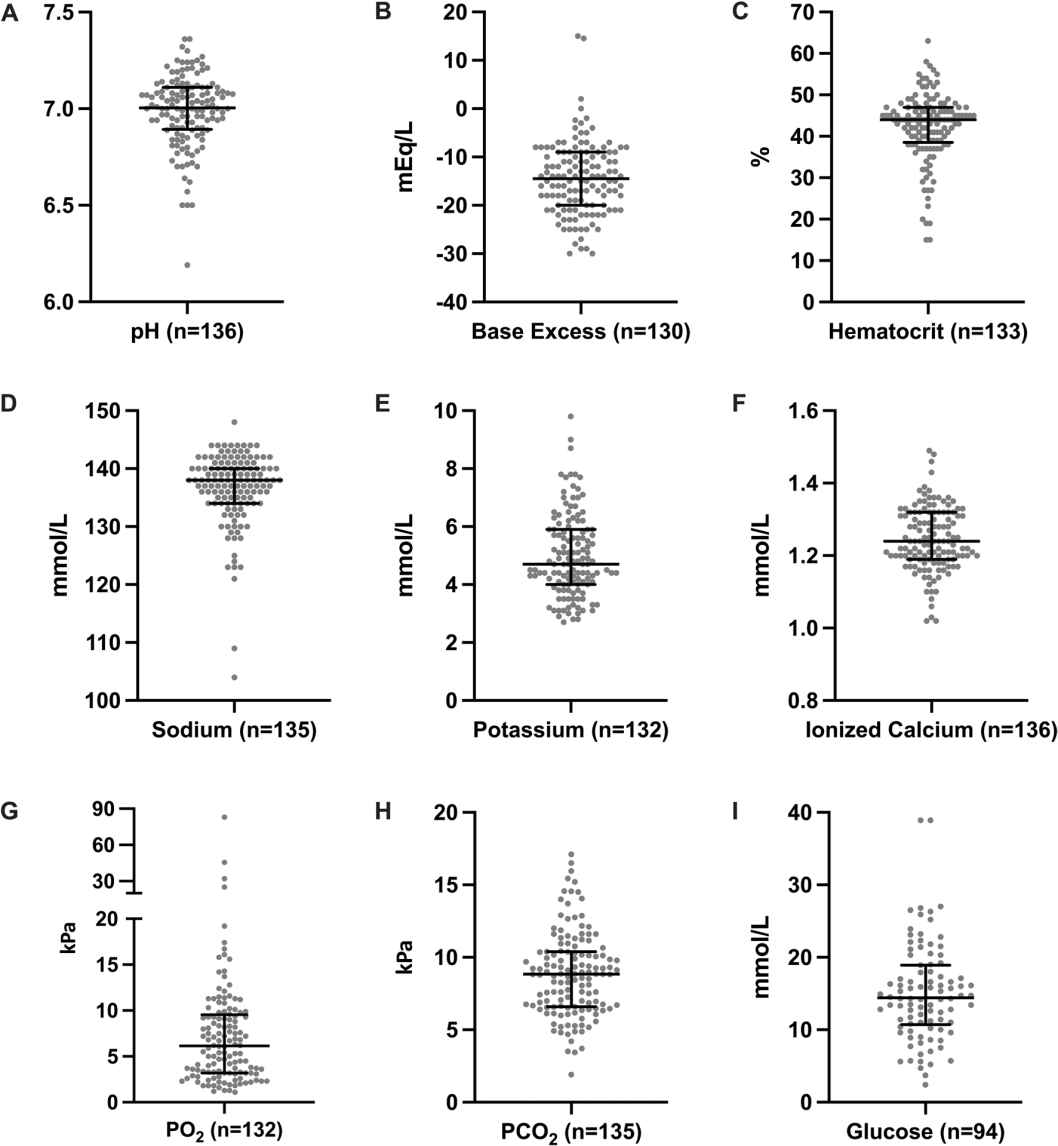
**A–I** Blood sample analysis during cardiac arrest. Lines show median and 25^th^–75th percentiles. The measurements exceeding the limits of analyse device are plotted on lower or upper limit

Among the 331 patients who were examined by POCUS, the documented findings are presented in Table 2. The POC blood sample analysis findings related to any possible reversible causes of OHCA (n = 136) are presented in Fig. 2. The most frequent laboratory disturbances were acidosis (n = 134) and hyperglycaemia (n = 86) (Fig. 2).

## Discussion

The main finding of the current study is that POCUS or laboratory analyses were used in 84% of the OHCA cases encountered by the HEMS crew, and both diagnostic tools were used in 18% of the cases. Furthermore, cause-specific treatment for the underlying primary cause was provided in approximately 15% of OHCA cases and even more frequently if diagnostic procedures had been performed.

We found that different pathologies related to potentially reversible causes of OHCA, such as hypovolaemia, dilatation of right ventricle or pericardial effusion, were screened with POCUS examination during resuscitation. This is consistent with previous studies, which have demonstrated the feasibility of ultrasound and its capability as a diagnostic tool during CPR [5, 15–19]. However, diagnosing pulmonary embolism by POCUS is highly unspecific method since the dilation of right ventricle can be caused by several other aetiologies demonstrated in animal studies [8, 9]. Further, the prognostic effects of identifying these pathologies with POC ultrasounds is unknown. The effectiveness of diagnostic tools is difficult to assess on scene and even more retrospectively. To our knowledge, only one study has shown that recognising these pathologies during CPR slightly improved patient outcomes [11]. However, commonly known confounders associated with survival from CA, e.g. age, sex, initial rhythm and bystander CPR, were not considered in the analyses [11]. Furthermore, others have yet to recognise the association between diagnostics and treatment to patient outcomes [20, 21]. The existence or absence of myocardial contraction, observed by POCUS examination, predicted short-term outcomes in two meta-analyses [22, 23].

Interpretation of POC blood sample analyses can be difficult in CA patients due to electrolyte changes caused by the CA (acidaemia and hyperkalaemia), and no specific regimen exists for this circumstance [1–4, 24]. Our work complements these findings. The most frequent POC blood sample findings in our study were acidosis (n = 134), hyponatraemia (n = 64), hyperglycaemia (n = 86) and hyperkalaemia (n = 57). Acidosis combined with hyperkalaemia is common during CA due to oxygen deficit in the tissues, leading to lactataemia [25]. A study of 263 OHCA patients receiving treatment for electrolyte abnormalities showed that treating these conditions were not consistent [11]. This indicates that even if diagnosing laboratory abnormalities during CPR is feasible, the treatment plans can vary and the effect on outcome is unknown.

Cause-specific treatment was provided infrequently in this study, and the benefits of the treatments remain unknown. The data concerning cause-specific treatment during CPR are controversial. Most of the treatment plans have yet to show an impact on outcome [21]. Still, there are some cause-specific treatments which have been shown to have a positive effect on outcome. ECPR is used for patients with recurrent VF who do not respond to CPR and defibrillation [1–4]. The benefit of ECPR has been shown in in-hospital CA however in OHCA patients the benefit is unclear [26–28]. In addition, the selection of patients for ECPR should be carefully considered in order to ensure better outcomes [29]. ECPR was also the most frequent cause-specific treatment in our study. Thrombolysis, the second most common specific intervention in the current study, is associated with good outcomes when given deliberately during OHCA [30, 31]. Administration of sodium bicarbonate (NaHCO_3_) during CA is not routinely recommended due to lack of evidence of benefits [1, 3, 32]. However, it may be beneficial in patients with hyperkalaemia or in certain poisonings [1]. In our study, NaHCO_3_ was administrated infrequently; however, the threshold for administering NaHCO_3_ was inconsistent between physicians. In conclusion, cause-specific treatment during CPR varies between physicians, and data concerning the benefits are scarce. It is uncertain when and to whom these treatments should be administered.

The previous resuscitation guidelines actively recommended searching for the underlying pathology and pointed out that POCUS and blood sample analyses can be used to screen these pathologies. The current guidelines advice against the routine use of POCUS [1–4]. However, no specific algorithm is available and the use of differential diagnostic tools is in the consideration of the individual physician operating. This can explain why in our study, both differential diagnostic tools were used only in 17% of the resuscitations with the involvement of a HEMS physician. In order to ensure the use of differential diagnostic tools a protocol is needed. When differential diagnostic tools were used, the patients were more likely to receive a specific treatment. This could indicate that differential diagnosis should be made more often during CPR to devise specific treatment plans. On the other hand, two studies which examined the underlying pathologies of CA demonstrated that a cardiac event (myocardial infarction, coronary artery disease or heart failure) was the leading pathology in about 60% of the patients and that other treatable causes were rare [21, 33]. The current treatment options for CA with a presumable cardiac aetiology are ECPR and percutaneous coronary intervention. The decision to implement ECPR is generally based on detecting recurrent VF rather than other differential diagnostics [1–4]. Furthermore, CA patients with a non-shockable initial rhythm were more likely to have a non-cardiac cause for the CA [34]. Thus, these patients could arguably benefit from active differential diagnostics using POC blood sample analyses and POCUS.

### Limitations and strengths

Our study has limitations that need to be considered when applying the results. First, due to the retrospective data collection, the data were partly incomplete and prone to reporting bias. Ultrasound and blood analysis findings were not always reported precisely, which limited data validity. In addition, the lack of POCUS protocol in the study unit does not allow the estimation of incidence of specific POCUS findings. Furthermore, even though blood samples were documented as arterial, accidental and undetected venous samples in some patients are possible effecting the blood analyses results presented in Fig. 2. Finally, differential diagnostics reaches far beyond ultrasound examination and POC blood sample analyses and should include, for example, the medical history and clinical examination of the patient. Because of the study design, we cannot evaluate these issues affecting the decision-making process. Second, the patient group which received cause-specific treatment was fairly small, reducing the ability to generalise the conclusions. Therefore, we did not collect outcome data in this study, hence the subgroups of patients were underpowered to reveal any clinically relevant differences.

Third, the results are possibly affected by selection bias because patients with ROSC before the HEMS unit’s arrival were not included, nor were the patients whose resuscitation attempt was immediately ceased by the HEMS unit because of a hopeless prognosis. Finally, the data were collected from only one unit, and thus, one should be cautious in generalising the results.

Nevertheless, our study covers OHCA cases with HEMS involvement from a population of 1.3 million people over 3.5 years. Consequently, the amount of data is high enough to represent the true incidences.

## Conclusions

In our study, HEMS physicians deployed POCUS or blood sample analyses in 84% of the encountered OHCA cases. Cause-specific treatment was administered in 15% of the cases. Our study demonstrates the frequent use of differential diagnostic tools though the rare administration of cause-specific treatment during OHCA. The feasibility and effectiveness of protocols for differential diagnostics during OHCA need to be evaluated in prospective trials.

## Data Availability

The dataset analysed during the current study is available from the corresponding author on reasonable request.

## Abbreviations

ALS: Advanced life support
CA: Cardiac arrest
CPR: Cardiopulmonary resuscitation
DNAR: Do not attempt to resuscitate
ECPR: Extracorporeal CPR
EMS: Emergency medical service
ERC: European Resuscitation Council
HEMS: Helicopter emergency medical service
LUCAS: Lund University Cardiopulmonary Assist System
NaHCO_3_: Sodium bicarbonate
OHCA: Out-of-hospital cardiac arrest
POCUS: Point-of-care ultrasound
ROSC: Return of spontaneous circulation
STROBE: Strengthening the reporting of observational studies in epidemiology
VF: Ventricular fibrillation
